# Exploring mental health stigma among Indonesian healthcare students towards individuals with mental illnesses: a qualitative study

**DOI:** 10.1080/17482631.2024.2327103

**Published:** 2024-03-11

**Authors:** Muhammad Arsyad Subu, Jacqueline Maria Dias, Richard Mottershead, Fatma Refaat Ahmed, Sari Narulita, Maryuni Maryuni, Zakiyah Zakiyah, Irma Nurbaeti, Alounoud Mohamed Al Marzouqi, Nabeel Al-Yateem

**Affiliations:** a Nursing department, College of Health Sciences University of Sharjah, Sharjah, United Arab Emirates; b Faculty of Nursing and Midwifery, University of Binawan, Jakarta, Indonesia; c Faculty of Health Sciences, UIN Syarif Hidayatullah, Jakarta, Indonesia; d Department of Health Service Administration, College of Health Sciences, University of Sharjah, Sharjah, United Arab Emirates

**Keywords:** Mental illness, stigma, students, campus, healthcare, qualitative

## Abstract

**Background:**

The social disapproval or stigma surrounding mental illness contributes to the postponement of individuals seeking assistance and frequently undermines therapeutic alliances between mental illness sufferers and health care professionals.

**Aims:**

This study explored perceptions and attitudes towards individuals with mental illness among college healthcare students in Indonesia.

**Methods:**

This study used a qualitative method. Twenty five students enrolled in six healthcare programs were interviewed using a semi structured format. The data analysis adopted a thematic analysis.

**Results:**

Our thematic analysis generated four main themes: (1) general perceptions of mental health and mental illness; (2) knowledge about mental illness; (3) mental health stigma; and (4) mental health stigma campaigns.

**Conclusions:**

The participants exhibited positive perceptions of mentally ill people. Students understood mental health, and they exhibited positive attitudes toward mentally ill people. Some students have stigma and lack of confidence to assist those who have mental illness. Further efforts are required to acquaint students with mental health issues and facilitate their interaction with mentally ill individuals. Anti-stigma campaigns are required to combat the pervasive stigmatization of individuals with mental illness. It is recommended to conduct a more extensive study about the stigma that students encounter in relation to mentally ill individuals.

## Introduction

The World Health Organization [WHO] (2022) estimates that one in every eight individuals worldwide is afflicted with a mental illness. A mental illness is distinguished by a substantial disruption in the cognition, affective regulation, or behaviour of the affected individual. It is frequently accompanied by distress or significant functional impairment. Mental illnesses are sometimes denoted as mental health conditions as well. The latter is a more inclusive designation that encompasses psychosocial disabilities, mental illnesses, and other mental states that are characterized by substantial distress, functional impairment, or the potential for self-harm (WHO, 2022). The majority of individuals with mental illness do not seek treatment due to concerns such as damaging their family’s reputation, diminishing marriage prospects, facing discrimination, social exclusion, and stigma (World Health Organization [WHO], 2019). Individuals who are afflicted with mental illness face an increased likelihood of encountering challenges such as poor academic achievement (Bruffaerts et al., 2018), and diminished self-esteem (Stuart et al., 2019).

Stigma can be described as the manifestation of prejudiced feelings and unfavourable conduct directed towards individuals who possess devalued attributes, which may be partially attributable to ignorance of claimed attributes (Corrigan, 2014). Stigma is a socially discrediting attribute or behaviour that causes an individual to be regarded as undesirable or atypical by the general public. Stigma is “an attribute that is deeply discrediting. It diminishes the social status of an individual by reducing them in the eyes of others from being whole and usual to tainted and discounted” (Goffman, 1963) (p. 3). Stigmatization has the potential to diminish an individual’s self-esteem, cause friction within familial units, and have an impact on their employability (Picco et al., 2019). Stigmatizing experiences of this nature have the potential to intensify an individual’s sense of exclusion and lack of competence, which in turn can have negative consequences for their motivation to seek treatment and maintain their engagement in treatment (Hadera et al., 2019).

Mental illness has historically been subject to social stigma, and individuals with mental illness are not regarded with the same level of societal respect as those without mental illness in any country, society, or culture (Rössler, 2016). Among Middle Eastern adolescents, stigma is believed to affect an estimated four out of every five individuals with mental illness (Mohammadzadeh et al., 2020). It is an action that engenders considerable anguish for those who encounter its repercussions on a daily basis (Link & Stuart, 2017). The stigma associated with mental health issues is pervasive; it is possible to find instances of prejudice or discrimination against those with mental health problems throughout every region of the globe (Seeman et al., 2016). Individuals with mental illness are generally perceived unfavourably by the general population (Al-Alawi et al., 2017). They are generally shunned, despised, and dreaded by society; this demonstrates the pervasiveness of stigmatization (Sousa et al. (2012). The stigmatization of individuals with mental illnesses is not an issue that is exclusively perceived by the common population. Surprisingly prevalent within the healthcare system and among healthcare professionals is stigmatization of mental health. Stigma associated with mental illness can manifest at the structural, interpersonal, and intrapersonal levels. Individuals with mental illnesses often express experiencing feelings of dehumanization, rejection, and devaluation when interacting with health professionals (Knaak, Mantler & Szeto, 2017). Stigma can be a significant factor in preventing individuals with mental health issues from receiving appropriate treatment. Due to the stigma associated with mental illnesses, a person may be reluctant to seek treatment for fear of being stigmatized or treated differently by society.

It was previously believed that healthcare professionals were impervious to these cognitive, emotional, and behavioural attacks against individuals with mental illness (Saridi et al., 2017). Stigmatizing perceptions of mental illness are not confined to uninformed members of the general public; even highly educated professionals across the majority of health disciplines hold such prejudices (Picco et al., 2019). Myths regarding the aetiology of mental illness serve to amplify the stigmatization of individuals with mental illness. Some Arabs, for instance, attribute its origin to malevolent spirits, black magic, or the evil eye (Alahmed et al., 2018; Merhej, 2019). Consequently, families frequently turn to Islamic scholars for assistance instead of mental health professionals, potentially resulting in treatment setbacks, treatment delays, or exacerbation of the individual’s condition (Al-Adawi, 2017; Merhej, 2019; Subu et al., 2023). Prior research has documented that education programmes have a substantial impact on adolescents’ knowledge and stigma reduction (Campos et al., 2018). An approach proposed to mitigate stigma, enhance help-seeking behaviours, and streamline access to treatment involves augmenting the overall mental health literacy of the general population (Huang et al., 2019).

### Research objectives

It is critical to explore perceptions and the stigmatizing attitudes exhibited by healthcare students towards these populations prior to developing educational interventions aimed at reducing stigmatizing behaviours and increasing awareness among these future professionals. Negative, stigmatizing attitudes among healthcare professionals can impede the development of therapeutic relationships and the provision of high-quality services (Radmanović & Burgić, 2017). This study explored perspectives and attitudes of healthcare students towards mental illnesses in Indonesia. Qualitative research enabled the collection of data while delving into contextual factors, recurring patterns, and unanticipated discoveries. This provided clarity regarding the most effective approaches to managing individuals with mental illnesses.

## Methods

### Research design

This study explored the perceptions and attitudes of healthcare students towards mental illnesses in Indonesia using a descriptive qualitative approach. To stay as near to the raw facts as possible, the research techniques used in qualitative description are inductive and result in a low-inference description of a phenomenon. The knowledge, attitudes, and behavioural responses of healthcare students in the setting of mental illness were analysed and identified using thematic analysis which seeks to reveal patterns, meaning, and themes (Braun & Clarke, 2006).

### Settings and participants

This study was conducted in the Faculty of Health Sciences University of Binawan Jakarta Indonesia. Through the use of class announcements, undergraduate healthcare students were recruited to participate in semi-structured interviews. A purposive sampling technique was employed to enlist twenty-five students from six healthcare programmes ((Pharmacy, Medical Laboratory Science, Nutrition, Physiotherapy, and Occupational Health & Safety. Faculty of Nursing and Midwifery) at the University of Binawan in Jakarta Indonesia to participate in the interviews. There were four students as participants represented in each of five healthcare disciplines (Pharmacy, Medical Laboratory Science, Nutrition, Physiotherapy, and Occupational Health & Safety. Faculty of Nursing and Midwifery represented five participants. An email was sent to each student who expressed interest in participating in the study by the researchers.

### Data collection

Data collection was conducted between June and August 2022. Semi structured interview is the main data collection method. Study participants were interviewed to better understand the context of perspectives and attitudes responses that healthcare students have towards mental illness. A set of preset questions were used in the semi-structured interview process. The interview guide included eight questions as follows:

1. Could you tell me a bit about yourself?
2. Could you tell me about your personal interactions and experiences with individuals dealing with mental health problems?
3. How knowledgeable are you regarding mental health and mental illnesses?
4. Could you explain your feelings of empathy and compassion towards individuals afflicted with mental illnesses?
5. In your opinion what you would do if you suffered from a mental illness?
6. What types of work experience have you gained since beginning your programme that involves individuals with mental illnesses?
7. Based on your personal and educational experiences as well as your understanding of mental illnesses, how would you describe your experience working with individuals who are with mental illness?
8. Is there anything else you can explain about mental illness and your personal as well as your learning experiences?

The interviews were conducted using the pre-planned interview questions, and they took 35 to 45 minutes. Participants were interviewed about a range of topics, including their past experiences and perspectives on various research components.

### Data analysis

The data analysis in this study was conducted in accordance with Braun and Clarke’s (2006) six-step thematic analysis. Through thematic analysis, pertinent patterns in the data that support the study’s objective were identified. During phase one (familiarization), an exact transcription was performed on each interview. Step two involved conducting line-by-line coding, wherein fundamental segments of information extracted from the unprocessed data were annotated and allocated codes (i.e., initial codes were generated). All codes were entered into a distinct spreadsheet during phase three (theme search), where they were evaluated and discussed by all authors to identify and group comparable codes into themes. Phase four (theme review) involved an assessment of the themes’ validity with respect to internal homogeneity. We met frequently during this phase to discuss the data in order to make any required modifications and revisions. We re-read all data during phase five (defining and naming themes) to verify that the themes were consistent with the data and to elucidate the relationships between the themes. Phase six, which entails report production, culminated in the creation of a document that presented the research findings. In order to maintain internal consistency and coherence among the data within categories and themes, the research team convened biweekly for discussions regarding the analysis procedure (Braun & Clarke, 2006). The themes were merged through a process that involved comparing the themes extracted from each data set, identifying, and discussing any inconsistencies that emerged, and negotiating and ultimately agreeing on themes that most faithfully represented the meanings of data. Then, each theme was defined in a way that captured and reflected the perceptions and attitudes of the students regarding mental illness.

### Study rigor

In this study, the rigour standards for qualitative data proposed by Chiovitti and Piran (2003) were implemented. Credibility, auditability, and fittingness were all components of this rigour criterion. Credibility was enhanced in this study by staying close to the data by word-by-word, line-by-line, and using in vivo coding, facilitated and reflected the participants’ experiences. During data analysis, we met regularly every two weeks to review all of the data collected. In addition, we discussed about the analysis procedure and other concerns that emerged in connection with the data acquisition and analysis processes. Auditability is the degree to which an additional researcher can perceive the researcher’s methods and reach the same conclusion. Auditability is enhanced by indicating the criteria used to formulate the researcher’s thinking, and by detailing how and why the participants in the study were chosen (Chiovitti & Piran, 2003). In this study, we carefully documented the research process and findings. All decisions were documented throughout the research process. Fittingness refers to the likelihood that the findings of a study can be extrapolated to analogous circumstances at large (Chiovitti & Piran, 2003). One can attain a sense of appropriateness by explicating the relationship between the literature and every category that emerged, as well as by defining the scope of the research in terms of the participants, location, and intensity of the themes generated. All coded data were evaluated by other authors during the coding process, providing an external validation of the findings. Another expert or researcher conducts an independent analysis of the data in order to validate the findings. In addition, an external researcher (IW) conducted an additional examination of all the coded data throughout the coding process.

### Ethical consideration

The research was carried out in adherence to the guidelines outlined in the Declaration of Helsinki. The research was granted approval by Research and Ethics Committee (REC) Universitas Binawan, Jakarta Indonesia (No: 108/EP/KE/UBINAWAN/VIII/2022). Once ethical approval was obtained, researchers approached prospective participants in each programme and provided them with an explanation of the study’s objectives, methodologies, and importance. Prospective participants were duly apprised that their involvement is discretionary, that they retain the right to disengage from the study at any moment, and that their choice to participate or not would not impact their subsequent academic pursuits. Prior to the interviews, each student was furnished with a comprehensive outline of the study’s objectives and duly informed that their involvement was entirely voluntary. Each participant was provided with a research information document which contained a comprehensive description of the study. Additionally, they were informed that they could withdraw from the study at any time. Incentives for student participation were not offered. Before beginning data collection, we obtained the written informed consent of participants and ensure the confidentiality and anonymity of their information. In order to prevent transcriptions from being associated with specific participants, a unique alpha-numeric designation was allocated to each individual (e.g., P1 denoting participant 1, P2 participant 2, and P3 participant 3 etc.). Each recorded interview was transferred to a password protected computer. The interview content, including both recordings and transcriptions, will be exclusively accessible to the researchers.

## Results

A total of 25 students participated in this study. Participants were from six different healthcare programmes at the University of Binawan Jakarta Indonesia. Of the total healthcare students participated, twelve were female and thirteen were male. They aged between 19 and 24 years old. Our thematic analysis generated four interrelated main themes: (1) General perceptions of mental health and mental illnesses; (2) Knowledge about mental illness; (3) Mental health stigma; and (4) Mental health stigma campaign. Furthermore, theme 1: General perceptions of mental health and mental illnesses has two subthemes (contact experience with mentally ill individuals and empathy with mentally ill individuals). Theme 2: Knowledge about mental illness has no subtheme. Theme 3: Mental health stigma has three subthemes (sources of mental health stigma, impacts of mental illness stigma, and fear towards people with mental illness). Theme 4: Mental health stigma campaign has no subtheme.

### Theme 1: general perceptions of mental health and mental illnesses

Subtheme: Contact experience with mentally ill individuals

The participants’ encounters with mental illnesses included personal experience, familial ties, or acquaintances with individuals who have struggled with mental illness in volunteer work, academic institutions, or workplaces. Some participants possessed clinical practice experience working with clients who were mentally ill patients. A nurse student said,

Having contended with mental health issues, I can speak from personal experience within the mental health system. … has exposed me to individuals with a variety of diagnoses, as have my family and close friends. (Participant 9).

Subtheme: Empathy with mentally ill individuals

Student participants asserted that mental illnesses were not prevalent in everyday life and that individuals with mental illness should be treated similarly to those with physical illnesses. An occupational health & safety student said,

… I believe we all possess this… This notion that we are obligated to provide a secure environment for the patient, even though the fact that they have a mental illness, is our responsibility. Furthermore, we must regard them as normal individuals … (Participant 15).

### Theme 2: knowledge about mental illness

It seemed that the participants possessed a basic knowledge of mental illnesses. Some causes of mental illness were recognized by the majority of participants, including genetic predisposition, social and environmental factors. Nevertheless, a considerable number of respondents expressed a lack of familiarity with managing patients who suffer from severe mental illnesses. One pharmacy student said,

While I am equipped with the means to recognize certain issues and consider them potentially problematic, I am far from being sufficiently educated to offer substantial assistance to others with mental illness. (Participant 15).

### Theme 3: mental health stigma

The majority of those who participated were aware of the social stigma associated with mental illness. According to them, negative societal attitudes towards mental illnesses continue to exist. A nurse student said,

It constitutes a social stigma. Indeed, numerous individuals perceived those with mental illnesses as unstable, unpredictable, confused, and threatening. It is true in our society. Yes, I am of the opinion that a stigma exists, but it is certainly diminishing, or so I hope. Stigma, in my opinion, no longer exists or is diminishing in our society. (Participant 10).

Subtheme: Sources of mental health stigma

Mental health problems were characterized as invisible or undetectable disabilities, which consequently posed a challenge to understanding. The participants recounted the frequent challenges they encountered in identifying symptoms of mental illnesses, particularly in differentiating between typical mental responses and those that would be classified as disorders. An occupational health & safety student said,

… I previously held the belief that my lack of initiative and resourcefulness was due to laziness, and that all I needed to do was exercise self-control, become motivated, and develop discipline…. It started from my own perception that he was inadequate. Also, I believe ignorance regarding mental illness is the source of stigma. In other words, one that promotes awareness of mental illness without advocating for those with mental illness was deemed to exacerbate the stigma associated with mental illness. (Participant 18).

Subtheme: Impacts of mental illness stigma

The participants in the study recognized that the stigma associated with mental illness had a substantial influence on different aspects of life. Mental health patients appeared to be perceived as weak, inadequate, and unprepared to deal with the challenges of daily life. Participants indicated that individuals with mental illness were frequently hesitant to reveal their condition to those in their immediate social circle. A physiotherapy student said,

… It is the term “social stigma”. It is acceptable for them to display weakness in the family and acquaintances, but in a society where individuals are evaluated based on their abilities…. They appear to be concealing the fact that they suffer from a mental illness. (Participant 10).

The stigma associated with mental illness was characterized as an impediment to forming and maintaining meaningful partnerships, causing feelings of isolation, disconnection, alienation, and exclusion. A nurse student said,

The lack of significant relationships between individuals with mental illness and without mental illnesses limits the ability to perceive things as they truly are and results in a lack of knowledge. The fear and confusion that ensue result in stigma. (Participant 15).

Subtheme: Fear towards people with mental illness

The dominant feeling among the participants was the presence of fear. Primarily, this attitude is referenced in relation to potential harm or unstable behaviours. An occupational health & safety student said,

People are fearful and me too. Yes, they [patients] must be hospitalized. Their fear prevents them from engaging in interactions with them [patients] normally. Then, it is better for their health and the individuals whose presence may influence their behavior that they be hospitalized. It is possible that I do not know precisely what will happen… Someone must treat them with care. (Participant 9).

### Theme 4: mental health stigma campaign

The study participants provided recommendations for enhancing their programmes, which comprised the following: placing a greater emphasis on mental health; incorporating teachings about mental illness earlier in the programme; increasing student exposure to clients with mental illnesses; and undertaking initiatives to destigmatize mental illness. An occupational health & safety student said,

It could be beneficial to have students engage with clients who have mental health issues right from the beginning of their program. It is the most important factor. (Participant 6).

Two strategies were highlighted by study participants as particularly effective in addressing the stigma associated with mental illness on campus. A physiotherapy student said,

To address the mental health stigma, it would be beneficial to provide situations that demonstrate the prevalence of such conditions, the ability of individuals to maintain a sense of normality … and offer guidance on appropriate coping mechanisms. (Participant 16).

Students must be educated about mental illness in order to combat the stigma associated with it. A health science and technology student said,

Mental health education is important … for example, campaign or awareness initiative… Promoting the acceptance and normalization of mental illnesses may reduce individuals’ sense of isolation and foster a greater propensity to seek assistance for these kinds of mental health problems. (Participant 23).

## Discussion

We found in this study that the participants possessed a rudimentary comprehension of mental illness and were able to identify a variety of treatments and causes of mental illness. Nevertheless, the study findings revealed the presence of several stigmatizing perceptions and attitudes. Individuals afflicted with severe mental illness are invariably perceived less favourably than those with physical illness. Individuals with mental illness are commonly stigmatized and excluded from society (Farina, 1998). We also found in this study that participants exhibited a more pessimistic perspective regarding the management of mental illness when compared to physical illness. This was ascribed by some participants to the imperceptibility and unpredictability of mental illness. Although the participants demonstrated knowledge of prevalent mental health concerns like anxiety and stress, a significant number of them reported having limited experience managing patients with severe mental illnesses. As a result, they lacked the readiness to help patients with mental illness. This study found that students’ interactions with people who had mental illnesses resulted in positive changes, including alterations in their worldview and perception and the growth of empathy. This is consistent with the finding that stigma can be reduced through direct contact with individuals who have mental illnesses (Nguyen et al., 2012). Some participants recounted the difficulties they encountered when a family member has mental illness, including the strain on the detrimental effects on familial relationships. This finding aligns with previous research that has documented comparable emotional strains and stressors experienced by those who offer assistance and care to a family member with mental illness (Shamsaei et al., 2015). The results and quality of mental health care are largely determined by the attitudes and knowledge of these professionals, primarily because they have a direct impact on resource allocation, treatment, communication, and the quality of care delivered; professionals lacking in these areas have the capacity to deliver substandard care (Jadhav et al., 2007). This study found that certain healthcare students maintain stigmatizing attitudes towards those with mental illness. Similarly, individuals who have mental illnesses often express experiencing feelings of being dehumanized, devalued, and disregarded when interacting with health professionals (Knaak et al., 2017). Mental health professionals have been found to hold these negative views (Wahl & Aroesty-Cohen, 2010).

In this study, participants noted that promoting a campus culture of self-care was identified as crucial to reducing the stigma associated with mental illness. A study conducted on medical students indicates that, in comparison to nursing students, their perspective on social treatment and the reintegration of individuals with mental illness is detrimental (Poreddi et al., 2014). Stuber et al. (2014) indicated that not all studies discovered adverse attitudes and behaviours among students and healthcare professionals towards individuals with mental illnesses. For example, health students under the age of 20 exhibit higher levels of tolerance, benevolence, and optimism. Students tend to categorize patients into distinct groups and exhibit less empathy towards those afflicted with mental illness in comparison to other users (Desai & Chavda, 2018). Healthcare practitioners who had more positive interactions with individuals who had mental illnesses held more receptive views regarding the civil rights of such individuals (Connaughton & Gibson, 2016; Henderson et al., 2014).

The majority of student participants are capable of recognizing and comprehending fundamental mental illness concepts. Similarly, Tognazzini et al. (2008) found that there may be a lack of education and awareness among healthcare professionals regarding mental illness. Supporting evidence suggests that mental health training may enhance the competencies and understanding of those who receive it, allowing them to assist those with mental illness more effectively (Hart et al., 2018; Rose et al., 2019). It is imperative that school and decision-makers consider these substantial associated factors and initiate efforts to enhance universities’ and colleges’ students’ understanding of mental illness. This can be accomplished by incorporating additional mental illness-related topics into the curriculum and hosting regular seminars for students. An additional efficacious intervention is social media content that promotes mental health (Halsall et al., 2019). Age and educational attainment were found to be positively correlated with knowledge of mental health (Kaushik et al., 2016). Some students develop more empathetic and less prejudiced attitudes towards individuals with mental illnesses after being exposed to such individuals (Anagnostopoulos & Hantzi, 2011).

Training in the acquisition of knowledge and beliefs that enhance the identification, management, and prevention of mental illness diminishes these prejudiced attitudes towards individuals with mental illnesses. This, in turn, results in a marginal improvement in treatment and an overall higher quality of life for affected patients (Lam et al., 2010). Similarly, research has shown that health professionals’ positive attitudes are enhanced when they have more than a month of experience providing psychiatric services (Hsiao et al., 2015). Understanding the beliefs and attitudes of medical students towards these users at an early stage is crucial for identifying potential negative perceptions and implementing appropriate measures to alleviate the stigmatization experienced by this group and its subsequent effects on the health care user population (Buechter et al., 2013).The international body of evidence indicates that healthcare students hold diverse perspectives regarding individuals with mental illness, which have a direct bearing on the quality of care and attention provided to those afflicted with such conditions (Stefanovics et al., 2016). This is crucial, as these attitudes have direct effects on the mental health of those affected, which can result in severe consequences like suicide (Campo-Arias & Herazo, 2015).

While there is an issue with stigma around mental illness among healthcare practitioners, it is less clear from the literature how students in healthcare education programmes see and perceive mental diseases (Amaechi et al., 2023). In this study, we found that the majority of student participants were aware of the social stigma associated with mental illness. Student participants indicated that mental illnesses are viewed negatively by society. Similar to this finding, stigma is increasingly concerned that healthcare personnel may not have the training and knowledge necessary to treat people with mental illnesses effectively (Tognazzini et al., 2008). Enhancing university students’ understanding of mental health contributes to the development of their mental health literacy (Rafal et al., 2018). According to our findings, the mental health education programme exhibits potential as an approach to enhance attitudes towards seeking assistance and diminish the negative perception surrounding mental illness. Knowledge regarding mental illness is crucial for increasing help-seeking and altering attitudes towards individuals with mental illnesses (Wei et al., 2013). People may be dissuaded from obtaining necessary treatment due to the adverse perception of mental health issues and the potential for shame associated with doing so (Gulliver et al., 2010). University students not only hold the perception that the general public endorses and sustains detrimental stereotypes regarding mental illnesses, but also exhibit similar behaviour (Lally et al., 2013).

The students in this study exhibited a reluctance to decelerate or interrupt their relentless pursuit of excellence, seemingly out of concern that doing so would result in the loss of advantageous opportunities that their peers were exploiting to advance. The social stigmatization of those with mental illness is a significant burden on those who are afflicted (Oexle & Corrigan, 2018). This research findings revealed that a subset of students recognized both favourable and derogatory societal perceptions of mental illnesses at the present time. Stigma is characterized by devaluation, exclusion, rejection, or guilt, and stems from the anticipation or experience of a negative social evaluation of an individual or group (Martin & Johnston, 2007; Subu et al., 2022). Similarly, mentally ill individuals often express sentiments of being devalued, neglected, and dehumanized during their interactions with healthcare professionals (Knaak et al., 2017). In addition, participants in this study exhibited attitudes that were stigmatizing, unsettling, and filled with dread regarding mental illnesses. Mentally ill individuals are among the most vulnerable populations, according to the evidence, because they are frequently subjected to discriminatory attitudes from the general public and healthcare professionals. These attitudes tend to limit their rights and produce disparities in health outcomes, treatment, and access (Poreddi et al., 2017). Due to these prejudices, discriminatory behaviours, and attitudes towards individuals with psychiatric illnesses, there is often a decline in quality of life, diminished treatment adherence, and a substantial contraction of social networks (Corrigan et al., 2013).

In this study, the student participants proposed a range of approaches to address the stigma associated with mental illness on campus. Some initiatives to reduce the stigma associated with mental illness in higher education have been developed. An approach that is practical and attainable by employing the existing methodologies could be utilized to mitigate the impacts of personal stigma on individuals’ inclination to seek health care (Al Omari et al., 2022). There has been a growing trend among universities to prioritize the promotion of students’ mental health (Conley et al., 2013). In addition, offering students direct interactions with individuals who are afflicted with mental illness can have a beneficial impact on the attitudes and conduct of adolescents (Yamaguchi et al., 2013). Stigma associated with mental illness may be diminished when students acknowledge and recognize their own mental health issues. Classes are more likely to produce positive mental health outcomes, such as reduced emotional distress, increased self-esteem, and enhanced social and emotional skills, when mental health prevention and promotion programmes are implemented with university students (Conley et al., 2013). In addition, awareness-raising efforts regarding mental health can encourage people to seek assistance when they require it and normalize the practice (Kutcher et al., 2015. The reduction of stigma and the enhancement of knowledge regarding accessible mental health services should be the focus of campus-based mental health awareness campaigns (Snyder, 2015). Campaigns promoting mental health awareness have the capacity to diminish the stigmatization of this issue among university students and encourage them to seek assistance. Furthermore, individuals who engaged in educational events demonstrated the greatest improvement in stigma attitudes (Funkhouser et al., 2017). In addition, enhanced understanding as well as diminished social stigma surrounding mental illness, contribute to the timely identification of such conditions, enhance mental health results, and improve mental health provisions (Rüsch et al., 2011). Campaigns promoting mental health awareness have been a tremendous success in altering attitudes and increasing awareness of mental health problems (Snyder, 2015). Despite the prevalence of mental health issues in school settings, the value of implementing specialized training among education professionals has not been studied to the same degree (Gallego et al., 2020; Wada et al., 2019). Stigma education were a required subject during the training of future professionals, such instruction would subsequently shape students’ perspectives (Wada et al., 2019).

### Study limitations

In this study, limited number of participants were selected from each of the six programmes for this research. Consequently, this study did not account for variations among the healthcare programmes. The study findings may not be applicable to other contexts due to the limited participants size, homogeneous characteristics, and university-specific nature of the study. The generalizability of the findings with respect to their representativeness of healthcare student populations and healthcare educational programmes is limited by the small-scale qualitative design. Further research may benefit from interdisciplinary variations that provide guidance for the development of mental health-specific curricula within healthcare programmes. Subsequent investigations may uncover strategies and interventions that diminish the social stigma associated with mental illness among healthcare programme students. Additional research is necessary to validate the findings of the present study within the context of Indonesia.

## Conclusions

The students participated in this study exhibited positive perceptions and attitudes towards mentally ill people. Students’ interactions with people who had mental illnesses resulted in positive changes and the growth of empathy. The majority of the participants reported experiencing positive effects as a result of interactions, leading to an enhanced understanding of the challenges associated with mental illnesses. The study findings revealed people with severe mental illness are invariably perceived less favourably than those with physical illness. The student participants possessed a rudimentary comprehension of mental illness. They were able to identify a variety of treatments and causes of mental illness. Although the participants demonstrated knowledge of prevalent mental health concerns like anxiety and stress, a significant number of students indicated that they have limited experience managing patients with severe mental illnesses. As a result, they lacked the readiness to help patients with mental illness. A significant proportion of the healthcare students maintained pessimistic perspectives on mental illness. With 25 participants, we found in this study that stigmatization of mental illness is pervasive in university settings. Study participants maintain stigmatizing perspectives towards individuals suffering from mental illnesses.

Further efforts are required to acquaint healthcare students with mental health issues and facilitate their interaction with mentally ill individuals. This was ascribed by some participants to the imperceptibility and unpredictability of mental illness. Anti-stigma campaigns and initiatives are required to combat the pervasive stigmatization of individuals with mental illness. It is strongly suggested that healthcare authorities, the public health sector, and higher education engage in interprofessional collaboration and operate across multiple domains—including the community, family, schools, and universities—in order to foster stigma-free mental health and raise awareness about stigma. Furthermore, it is recommended to conduct a more extensive investigation into the stigma that healthcare students encounter in relation to individuals who have mental illness.
